# Heterogeneity in pineapple fruit quality results from plant heterogeneity at flower induction

**DOI:** 10.3389/fpls.2014.00670

**Published:** 2014-12-09

**Authors:** V. Nicodème Fassinou Hotegni, Willemien J. M. Lommen, Euloge K. Agbossou, Paul C. Struik

**Affiliations:** ^1^Crop Physiology, Centre for Crop Systems Analysis, Wageningen UniversityWageningen, Netherlands; ^2^Faculté des Sciences Agronomiques, Université d'Abomey CalaviCotonou, Benin

**Keywords:** *Ananas comosus*, D-leaf, fruit size, variation, variation in quality, variation within crop, vigor

## Abstract

Heterogeneity in fruit quality constitutes a major constraint in agri-food chains. In this paper the sources of the heterogeneity in pineapple in the field were studied in four experiments in commercial pineapple fields. The aims were to determine (a) whether differences in pineapple fruit quality among individual fruits are associated with differences in vigor of the individual plants within the crop at the time of artificial flower induction; and (b) whether the side shoots produced by the plant during the generative phase account for the fruit quality heterogeneity. Two pineapple cultivars were considered: cv. Sugarloaf and cv. Smooth Cayenne. Plant vigor at the time of artificial flower induction was measured by three variates: the number of functional leaves, the D-leaf length and their cross product. Fruit quality attributes measured at harvest time included external attributes (weight and height of fruit, infructescence and crown) and internal quality attributes [total soluble solids (TSS), pH, translucent flesh]. Results showed that the heterogeneity in fruit weight was a consequence of the heterogeneity in vigor of the plants at the moment of flower induction; that effect was mainly on the infructescence weight and less or not on the crown weight. The associations between plant vigor variates at flower induction and the internal quality attributes of the fruit were poor and/or not consistent across experiments. The weight of the slips (side shoots) explained part of the heterogeneity in fruit weight, infructescence weight and fruit height in cv. Sugarloaf. Possibilities for reducing the variation in fruit quality by precise cultural practices are discussed.

## Introduction

In the last decades, customers have become more demanding on uniformity of agricultural products, in addition to quantity, quality and delivering time (Beamon, 1999). In pineapple [*Ananas comosus* (L.) Merrill] production, a large heterogeneity in pineapple quality (size and taste) is an important constraint for successfully meeting market requirements (Takane, 2004; Vagneron et al., 2009; Fassinou Hotegni et al., 2014). For export of agricultural products, the Codex Alimentarius (2005) has set a number of quality criteria; for pineapple these include the degree of acceptable fruit quality as well as the associated heterogeneity in fruit weight, fruit height, the ratio crown height: infructescence height, the total soluble solids (TSS) and percentage of damage. The heterogeneity in quality of a product is caused by many factors, including the cultural practices underlying its production (Luning and Marcelis, 2006; Ritter et al., 2008). Finding the source of product heterogeneity in the field is therefore fundamental for designing methodologies to obtain a more uniform product quality at harvest.

In pineapple, the high heterogeneity in quality at harvest may originate from a large heterogeneity in the vigor of the individual plants within a crop, especially at the time of flower induction. Pineapple is a vegetatively propagated, perennial crop, showing three partly overlapping phases: the vegetative phase, characterized by an increase in number of leaves and diameter of the main stem (from planting to flower induction); the generative phase (from flower initiation to fruit maturity); and the propagative phase when different types of side shoots are produced (starting during the generative phase and continuing after the fruit harvest). Different types of vegetative organs are used as planting material: slips (shoots produced on the peduncle at the base of the fruit), hapas or side shoots (shoots produced above ground on the stem at the junction of the stem and the peduncle), suckers (side shoots originating below ground from the stem) and crowns (produced at the top of the fruit) (Hepton, 2003) with slips, hapas, and suckers being the most frequently used planting material. Plants are single-stemmed in the first year of production. To proceed from the vegetative to the reproductive phase, growth regulators are applied that release ethylene or acetylene which induce and synchronize flowering of the main stem (Collins, 1960). This artificial flower induction takes place 6–16 months after planting depending on the environment (Malézieux et al., 2003) and the desired delivery time of the fruits (generally 5–6 months after flower induction) (Kerns et al., 1936; Bartholomew et al., 2003). After flower induction, the formation of vegetative leaves on the main stem ceases (Bartholomew and Malézieux, 1994) as the result of the transition of the apex to the generative stage (Bartholomew et al., 2003) and multiple florets are initiated at the apex. Vegetative leaf production is resumed later when the production of florets ceases and the crown leaves are initiated (Bartholomew et al., 2003). The stage of development of a crop at flower induction affects the later fruit weight, with a high number of leaves leading to larger fruits (Van Overbeek, 1946; Py and Pelegrin, 1958; Mitchell, 1962; Py and Lossois, 1962; Malézieux, 1993). Consequently, also the heterogeneity in fruit weight of the plants within a field may be related to the heterogeneity among plants at the time of flower induction. In some cultivars (e.g., Smooth Cayenne), fruit maturity is synchronized by applying the compound Ethephon (Smith, 1991).

A pineapple fruit consists of the infructescence and the crown. It is thus far unknown if and how their individual weights and height, and the ratio between crown and infructescence height are affected by the plant status at the time of artificial flower induction.

Defoliation of pineapple plants 3 weeks before harvest was shown to reduce the TSS concentration in the fruit and the fruit flesh translucency; the lowest values were obtained when all leaves were removed (Chen and Paull, 2000). This shows that the plant status can affect also internal fruit characteristics. It is thus far unknown if fruits from more vigorous plants at the time of flower induction, will show a different internal quality, e.g., a higher concentration of TSS, different juice pH, more translucent flesh, or different internal browning, when compared to fruits from less vigorous plants.

Also production of slips or other side shoots by the plant during fruit development may account for fruit quality heterogeneity. The initiation of slips occurs before the end of flower initiation (Kerns et al., 1936). Studies on the relation between slip pruning and the fruit size show contradictory results. Norman (1976) found that removing slips increased fruit weight; recent studies on the other hand revealed that slips were important sources of assimilates for fruit growth and maintenance (Marler, 2011a). Because the production of the slips overlaps with fruit development and growth, they may compete for input of assimilates from the leaves on the main stem. Therefore, the number and/or the weight of the additional vegetative organs produced might contribute—in addition to the plant vigor at flower induction—to the differences in fruit quality at harvest.

The objectives of this study were to analyze (a) if and how differences in quality attributes between individual fruits within a crop are associated with differences in vigor of the individual plants within the crop at the time of artificial flower induction; and (b) if and how the number and the weight of side shoots formed during the generative phase also account for fruit quality heterogeneity at harvest time in addition to the initial plant vigor at flower induction. Results will help to understand why fruit quality is variable and will allow development of precise cultural practices that will reduce the fruit quality heterogeneity at harvest.

## Material and methods

### Experimental site and design

Four on-farm experiments were carried out on commercial pineapple fields in the Atlantic department (characterized by a subequatorial climate) in the south of Benin (West Africa) between February 2010 and August 2012 with two pineapple cultivars: Sugarloaf (Experiments 1 and 2) and Smooth Cayenne (Experiments 3 and 4). Two different producers were selected per cultivar based on (a) the age of their pineapple crop being close to the common artificial flower induction time and (b) whether they applied the common practices for these cultivars, as described by Fassinou Hotegni et al. (2012). Pineapple cultivation starts with the planting materials obtained from harvested plants kept in the fields. During the planting material collection, those shoots that are very small or very big or look unhealthy are skipped; different types (hapas and suckers in cv. Smooth Cayenne) and sizes are mixed when planting. Information on the fields and cultural practices until artificial flower induction time is provided in Table 1.

**Table 1:** **Field information and cultural practices in the four experiments with cvs Sugarloaf or Smooth Cayenne**.

**Field information and cultural practices**	**Cv. Sugarloaf**		**Cv. Smooth Cayenne**	
	**Experiment 1**	**Experiment 2**	**Experiment 3**	**Experiment 4**
Location	06°36′09.2″N and 02°16′31.6″E	06°37′26.4″N and 02°16′13.1″E	06°36′43.7″N and 02°19′55.1″E	06°36′44″N and 02°19′54.3″E
Municipality (district)	Zè (Tangbo Djevie)	Idem	Abomey-Calavi (Zinvié)	Idem
Soil type (U.S. equivalent)	Ferralitic soil (Ultisols)	Idem	Idem	Idem
Planting time^a^	February 2010	July 2010	April 2011	May 2011
Type of planting material used^a^	Slips	Idem	Hapas and suckers	Idem
Planting material treatment before planting^a^	No treatment	Idem	Idem	Idem
Planting arrangement	Flat beds of two alternating rows	Idem	Idem	Idem
Plant spacing: BP^b^ × BR^c^/BDR^d^ (cm)	40 × 50/80	35 × 45/65	47 × 55/75	Idem
Plant density (plants/m^2^)	3.85	5.19	3.27	Idem
First Urea (46N) + NPK (10-20-20)	7 MAP^e^ (18 September 2010)	2 MAP (15 September 2010)	3 MAP (20 July 2011)	2 MAP (17 July 2011)
Application form	Solid at the base of the plants	Idem	Idem	Idem
Dose per plant (g Urea + g NPK)	6 + 3	Idem	5 + 4	Idem
Second Urea (46N) + NPK (10-20-20)	Not applied	Idem	6 MAP (15 October 2011)	5 MAP (24 October 2011)
Application form			Solid at the base of the plants	Idem
Dose per plant (g Urea + g NPK)			4 + 5	Idem
NPK (10-20-20) application	12 MAP (22 February 2011)	9 MAP (16 April 2011)	Not applied	Idem
Application form	Solid	Idem		
Dose per plant (g Urea + g NPK)	7	Idem		
K_2_SO_4_ (50-18)application	Not applied	Idem	10 MAP (8 February 2012)	9 MAP (17 February 2012)
Application form			Solid at the base of the plants	Idem
Dose per plant (g Urea + g NPK)			7	Idem
Artificial flower induction time	13 MAP (6 March 2011)	10 MAP (4 May 2011)	10 MAP (22 February 2012)	Idem (3 March 2012)
Weed control	Hand weeding	Idem	Idem	Idem
Harvest time	18 MAP (3–4 August 2011)	15 MAP (2, 3, and 5 October 2011)	15 MAP (24–25 July 2012)	Idem (3–4 August 2012)

a Information gathered from pineapple producer (field owner);

b BP, spacing between plants within a row;

c BR, spacing between rows;

d BDR, spacing between double rows;

e *MAP, months after planting*.

Four experimental plots were installed per experiment, which were part of a larger experiment not reported on here. Each net plot consisted of six rows of 10 plants each. The net experimental plots were surrounded by two rows with border plants.

### Artificial flower induction and maturity synchronization

Crops were artificially induced between 10 and 13 months after planting (Table 1) using carbide of calcium (CaC_2_), a compound producing acetylene when it reacts with water. Following farmer's practices, 50 ml of a solution containing 10 g/l and 15 g/l of CaC_2_ for Sugarloaf and Smooth Cayenne, respectively, was applied into the center of the leaf rosette of each plant. This application was carried out once in cv. Sugarloaf and three times, with an interval of 3 days, in cv. Smooth Cayenne.

Following farmer's practices, maturity of the fruits was synchronized only in cv. Smooth Cayenne, 143 days after artificial flower induction, by spraying 3.5 ml of a solution of 14 ml/l Ethephon (2-chloroethylphosphonic acid), a compound producing ethylene, on the skin of each fruit. This application was carried out twice with an interval of 4 days.

Pineapple fruits were harvested between 150 and 154 days after flower induction. The pineapple fruits were harvested following farmer's practice which was in cv. Sugarloaf the moment when the skin color of the fruit of at least 25% of the plants (i.e., 15 out of 60 plants in a net plot) had started to change from green to yellow. In cv. Smooth Cayenne, the fruits were harvested 7 days after the second application of Ethephon. All fruits per plot were harvested on that day and were individually processed. No ratoon crops are grown in Benin, so plants were harvested once.

### Observations and measurements

Three variates representing the vigor of the individual plants within a crop at the moment of artificial flower induction were assessed: (1) the number of functional leaves per plant (NL) (green leaves excluding those withered over more than 10 cm of their length), (2) the length of the D-leaf (DL) (the longest leaf in a pineapple plant according to Malézieux et al. (2003)) and (3) their cross product (NL × DL). The number of functional leaves indicates the developmental status of the plant at flower induction time. The D-leaf is used to assess the growth and the nutritional status of the plant (Malézieux et al., 2003). The cross product NL × DL is a proxy for the total leaf area of the plant. The number of functional leaves and DL were assessed on all individual plants 1 day before flower induction. The D-leaf was identified by bunching all leaves together and selecting the longest. Next, the length was measured with a twig combined with a ruler.

External and internal fruit quality attributes were assessed at harvest on the fruits from all individual plants. External fruit quality attributes included the weight and height of the fruit, infructescence and crown; the ratio crown height: infructescence height and the number of fruitlets per infructescence. The number of fruitlets or “eyes” on the infructescence was determined by multiplying the number of spirals counted counter-clockwise and the average number of fruitlets on the first and last spiral. Internal fruit quality attributes included TSS, juice pH, the percentage of flesh being translucent, and internal browning. To determine these, the pineapple was cut longitudinally into two halves. A portion of the juice obtained from squeezing one half was used to determine the TSS by a hand refractometer; another portion of that juice was used to determine the juice pH by a hand-held pH meter. The percentage of fruit flesh that was translucent and internal browning were visually estimated on the second half following the methods of Paull and Reyes (1996). The type, number and total weight of side shoots (slips, hapas, and suckers) per plant were also recorded at harvest time.

### Statistical analysis

Data were analyzed using R version 2.15.2 (R Development Core Team, 2012). Fruits with more than one crown at harvest (13 and 6 fruits in Experiments 3 and 4, respectively) were excluded in the analysis. Heterogeneity in plant vigor variates and in fruit quality attributes was described by the coefficient of variation (CV) which is a measure of the variability in a population relative to the mean (cf. Schouten et al., 1997; Illipronti et al., 2000; Field, 2009; Ott and Longnecker, 2010). CVs were calculated per plot and differences in CV between cultivars for each plant vigor variate and each quality attribute were assessed using a *t*-test. Differences in CV between plant vigor variates as well as differences in CV between quality attributes within an experiment were assessed using an ANOVA. When the *F*-value from the ANOVA was significant, LSDs (α = 0.05) were used to separate means.

To determine if and how the plant vigor variates at flower induction were associated with fruit quality attributes at harvest, simple linear regressions were performed on the combined data from all plots per experiment, using NL, DL, and NL × DL as explanatory variates and each fruit quality attribute as response variate. Percentage flesh translucency was transformed using square root transformation ($\sqrt{x+0.5}$) before analysis (Bartlett, 1936; Gonzalez, 2009). Which plant vigor variable was best associated with a fruit quality attribute was determined using the adjusted *R*^2^. The higher the adjusted *R*^2^, the higher is the percentage of the variance in the response variate accounted for.

To determine whether the number and the weight of the additional side shoots produced (slips) accounted for fruit quality heterogeneity in addition to the plant vigor variates at flower induction, a multiple regression was performed by using the plant vigor variates (explaining the highest percentage of the variance in the fruit quality attributes variates) as well as the number or weight of the slips as explanatory variates and the different fruit quality attributes as response variates. A hierarchical method was used in which the plant vigor variates were entered first and the weight or number of slips was entered next, to analyze the contribution of slip weight/number to fruit quality heterogeneity. Existence of colinearity between the explanatory variates was checked using Pearson coefficient of correlation (*r*). A value of *r* greater than 0.80 reveals multiple colinearity between the explanatory variates (Field, 2009); in that case the explanatory variables were not used in the multiple regression model. The significance of the *F* change (significance of the improvement of the adjusted coefficient of multiple regression *R*^2^) derived from the multiple regression model was used to evaluate the effect of slip weight/number.

## Results

### Initial heterogeneity in plant vigor at flower induction

The initial heterogeneity in plant vigor (NL, DL, and NL × DL) within a field was quantified using the CV. For all vigor variates, the initial heterogeneity was not different between experiments with cv. Sugarloaf and experiments with cv. Smooth Cayenne (Table 2). In all four experiments, variation in NL × DL was higher than variation in NL and DL, and variation in DL was lowest (Table 2).

**Table 2:** **Differences in plant vigor and fruit quality variation (CV) within experiments and between experiments with different cultivars**.

**Variates**	**Cv. Sugarloaf**		**Cv. Smooth Cayenne**		**Difference between cultivars**	
	**Experiment 1**	**Experiment 2**	**Experiment 3**	**Experiment 4**	***P*-value^a^**
**PLANT VIGOR VARIATES AT THE TIME OF FLOWER INDUCTION**					
Number of functional leaves (NL)	0.21 b^b^	0.24 b	0.22 b	0.26 b	0.308
D-leaf length (DL)	0.12 a	0.08 a	0.09 a	0.09 a	0.225
NL × DL	**0.28 c**	**0.29 c**	**0.26 c**	**0.33 c**	0.630
Difference within experiments: *P*-value^c^	0.000^***^	0.000^***^	0.000^***^	0.000^***^	
**EXTERNAL FRUIT QUALITY ATTRIBUTES AT HARVEST**					
Fruit weight	0.33 c	0.28 d	0.30 c	0.34 e	0.535
Infructescence weight	**0.39 d**	**0.33 e**	**0.38 d**	**0.42 f**	0.087
Crown weight	0.24 b	0.18 b	0.31 c	0.27 d	0.007^**^
Fruit height	0.11 a	0.09 a	0.13 a	0.11 a	0.167
Infructescence height	0.23 b	0.18 bc	0.20 b	0.21 bc	0.934
Crown height	0.13 a	0.11 a	0.20 b	0.17 b	0.000^***^
Ratio crown: infructescence height	0.31 c	0.22 c	0.32 c	0.32 e	0.039^*^
Number of fruitlets	0.25 b	0.18 b	0.20 b	0.23 cd	0.913
Difference within experiments: *P*-value	0.000^***^	0.000^***^	0.000^***^	0.000^***^	
**INTERNAL FRUIT QUALITY ATTRIBUTES AT HARVEST**					
Total soluble solids	0.06 a	0.06 a	0.10 a	0.10 a	0.000^***^
Juice pH	0.05 a	0.05 a	0.03 a	0.03 a	0.001^***^
Translucent flesh^d^	**0.81 b**	**0.70 b**	**2.39 b**	**1.16 b**	0.020^*^
Difference within experiments: *P*-value	0.000^***^	0.000^***^	0.007^**^	0.000^***^	

* Significant at the 0.05 probability level;

** Significant at the 0.01 probability level;

*** Significant at the 0.001 probability level;

a Assessed by a t-test;

b Within columns and groups of variates, values followed by the same letter are not significantly different according to the LSD (0.05);

c Assessed by ANOVA;

d *untransformed data used*.

*Values in bold indicate where the variation was the highest for each group of variates: plant vigor; external fruit quality attributes and internal fruit quality attributes*.

### Heterogeneity in fruit quality attributes at harvest

When comparing the CV in different external fruit quality attributes at harvest across experiments with different cultivars (Table 2), the variation in crown weight, crown height and ratio crown height: infructescence height was higher in the experiments with cv. Smooth Cayenne than in those with cv. Sugarloaf, whereas the variation in all other attributes was similar across cultivars.

In all experiments, variation in infructescence weight was higher than variation in other external quality attributes. Variation in fruit weight, infructescence weight and the crown weight was higher than in the respective heights of these organs in all experiments (Table 2). Variation in infructescence weight was higher than variation in fruit and crown weight. The crown weight was the least variable weight attribute except in Experiment 3, where it was comparable to fruit weight (Table 2). Variation in infructescence height was higher than variation in fruit height in all experiments (Table 2), whereas variation in crown height was comparably low as variation in fruit height in the Sugarloaf experiments and comparably high as variation in infructescence weight in the Smooth Cayenne experiments. Variation in the ratio crown: infructescence height was higher than that in the underlying attributes, except in Experiment 2 where the difference with the variation in infructescence height was not significant. The CV in number of fruitlets was similar to the CV in infructescence height.

For all internal quality attributes, variation in TSS and translucent flesh was higher in the experiments with cv. Smooth Cayenne than in the experiments with cv. Sugarloaf. Variation in juice pH was higher in experiments with cv. Sugarloaf than in experiments with cv. Smooth Cayenne. In all experiments, the most variable internal quality attribute was flesh translucency. Variation in TSS and variation in juice pH were very low and not significantly different from each other in all experiments (Table 2).

### Associations between plant vigor at the time of artificial flower induction and external fruit quality at harvest

**Figure 1:**
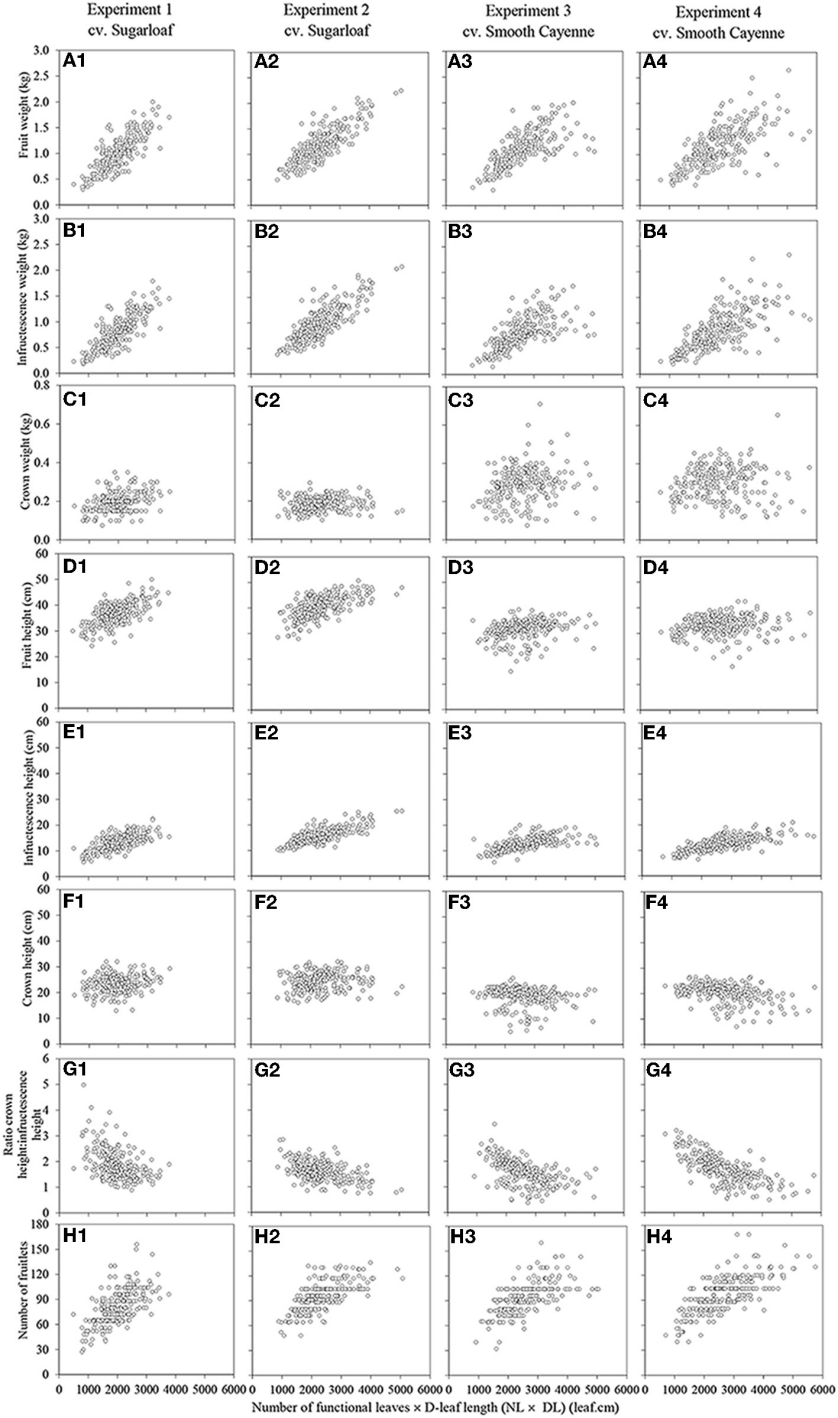
**Associations between the number of functional leaves × the D-leaf length (NL × DL) and the external fruit quality attributes in Experiments 1 (A1–H1) and 2 (A2–H2) (cv. Sugarloaf) and Experiments 3 (A3–H3) and 4 (A4–H4) (cv. Smooth Cayenne)**.

In all crops there were strong associations between the initial vigor of a plant at flower induction and the total fruit weight of that plant at harvest; higher NL, DL, and NL × DL all were associated with heavier fruits at harvest (Table 3). Based on adjusted *R*^2^ values (0.463 – 0. 686), NL × DL was the vigor variate showing the strongest association with fruit weight (Table 3; Figures 1A1–A4). The *R*^2^ values for the relations between plant vigor variates and infructescence weights were comparable to those for total fruit weights and also highest for NL × DL (Table 3; Figures 1B1–B4). However, *R*^2^ values for the relations between vigor variates and crown weight were much lower and not significant for NL × DL in two out of four experiments (Table 3; Figures 1C1–C4), suggesting that the positive associations between NL × DL and fruit weight were mainly caused by the positive effect of high vigor on the infructescence weight, and less or not on crown weight. Variation in crown weight was better explained by DL than by NL × DL, but with low *R*^2^ values varying between 0.024 and 0.142.

**Table 3:** **Linear regression models of the association between plant vigor variates [number of functional leaves (NL), D-leaf length (DL), number of functional leaves × D-leaf length (NL × DL)] at artificial flower induction (explanatory variates) and fruit quality attributes at harvest (response variates) across individual plants in the four experiments, cvs Sugarloaf and Smooth Cayenne**.

**Fruit quality attributes at harvest**	**Plant vigor at flower induction**	**Cv. Sugarloaf**				**Cv. Smooth Cayenne**			
		**Experiment 1 (*n* = 240)**		**Experiment 2 (*n* = 240)**		**Experiment 3 (*n* = 227)**		**Experiment 4 (*n* = 234)**	
		***R*^2^ adj**.	**Equation**	***R*^2^ adj**.	**Equation**	***R*^2^ adj**.	**Equation**	***R*^2^ adj**.	**Equation**
**EXTERNAL QUALIT*Y* ATTRIBUTES**									
Fruit weight	NL^a^	0.472	*Y* = −0.064 + 0.046^***^ NL	0.618	*Y* = 0.144 + 0.040^***^ NL	0.411	*Y* = 0.293 + 0.025^***^ NL	0.415	*Y* = 0.253 + 0.028^***^ NL
	DL^b^	0.500	*Y* = −0.839 + 0.022^***^ DL	0.282	*Y* = −0.685 + 0.021^***^ DL	0.196	*Y* = −0.496 + 0.020^***^ DL	0.297	*Y* = −0.913 + 0.025^***^ DL
	NL × DL	0.645	*Y* = 0.085 + 4.6.10^−4^^***^ NL × DL	0.686	*Y* = 0.280 + 3.9.10^−4^^***^ NL × DL	0.467	*Y* = 0.339 + 3.0.10^−4^^***^ NL × DL	0.463	*Y* = 0.370 + 2.9.10^−4^^***^ NL × DL
Infructescence weight	NL	0.474	*Y* = −0.194 + 0.043^***^ NL	0.617	*Y* = −0.033 + 0.040^***^ NL	0.410	*Y* = 0.062 + 0.023^***^ NL	0.464	*Y* = −0.041 + 0.028^***^ NL
	DL	0.482	*Y* = −0.890 + 0.020^***^DL	0.266	*Y* = −0.806 + 0.020^***^ DL	0.173	*Y* = −0.587 + 0.018^***^ DL	0.280	*Y* = −1.031 + 0.023^***^ DL
	NL × DL	0.638	*Y* = −0.048 + 4.3.10^−4^^***^ NL × DL	0.679	*Y* = 0.106 + 3.8.10^−4^^***^ NL × DL	0.458	*Y* = 0.110 + 2.7.10^−4^^***^ NL × DL	0.501	*Y* = 0.087 + 2.9.10^−4^^***^ NL × DL
Crown weight	NL	0.078	*Y* = 0.131 + 0.003^***^ NL	0.000	*Y* = 0.178 + 2.5.10^−4NS^NL	0.023	*Y* = 0.233 + 0.002* NL	0.000	*Y* = 0.294 + 0.0002^NS^NL
	DL	0.142	*Y* = 0.051 + 0.002^***^ DL	0.024	*Y* = 0.125 + 0.001** DL	0.037	*Y* = 0.092 + 0.002** DL	0.052	*Y* = 0.118 + 0.002^***^ DL
	NL × DL	0.133	*Y* = 0.135 + 3.0.10^−5^^***^ NL × DL	0.003	*Y* = 0.176 + 4.0.10^−6NS^NL × DL	0.034	*Y* = 0.230 + 2.3.10^−5^^**^ NL × DL	0.002	*Y* = 0.283 + 6.8.10^−6NS^NL × DL
Fruit height	NL	0.252	*Y* = 26.886 + 0.424^***^ NL	0.276	*Y* = 31.605 + 0.340^***^ NL	0.044	*Y* = 28.095 + 0.104** NL	0.007	*Y* = 31.644 + 0.043^NS^NL
	DL	0.402	*Y* = 15.800 + 0.249^***^ DL	0.377	*Y* = 13.080 + 0.310^***^ DL	0.035	*Y* = 22.912 + 0.107** DL	0.093	*Y* = 21.990 + 0.136^***^ DL
	NL × DL	0.402	*Y* = 27.599 + 0.005^***^ NL × DL	0.390	*Y* = 31.789 + 0.004^***^ NL × DL	0.060	*Y* = 28.020 + 0.001^***^ NL × DL	0.024	*Y* = 31.193 + 0.001*NL × DL
Infructescence height	NL	0.336	*Y* = 5.201 + 0.351^***^ NL	0.510	*Y* = 7.604 + 0.318^***^ NL	0.340	*Y* = 7.001 + 0.175^***^ NL	0.484	*Y* = 5.982 + 0.208^***^ NL
	DL	0.423	*Y* = −2.050 + 1.183^***^ DL	0.278	*Y* = −0.374 + 0.184^***^ DL	0.177	*Y* = 1.023 + 0.145^***^ DL	0.351	*Y* = −2.744 + 0.188^***^ DL
	NL × DL	0.476	*Y* = 6.218 + 0.004^***^ NL × DL	0.584	*Y* = 8.570 + 0.003^***^ NL × DL	0.401	*Y* = 7.218 + 0.002^***^ NL × DL	0.540	*Y* = 6.843 + 0.002^***^ NL × DL
Crown height	NL	0.010	*Y* = 21.685 + 0.074^NS^NL	0.000	*Y* = 24.001 + 0.022^NS^NL	0.021	*Y* = 21.094 − 0.070* NL	0.171	*Y* = 25.662 − 0.164^***^ NL
	DL	0.047	*Y* = 17.850 + 0.066^***^ DL	0.095	*Y* = 13.454 + 0.126^***^ DL	0.001	*Y* = 21.889 − 0.039^NS^DL	0.011	*Y* = 24.657 − 0.052^NS^DL
	NL × DL	0.031	*Y* = 21.381 + 0.001^**^ NL × DL	0.011	*Y* = 23.219 + 0.001^NS^NL × DL	0.021	*Y* = 20.803 − 0.001^*^ NL × DL	0.141	*Y* = 24.350 − 0.001^***^ NL × DL
Ratio crown height:	NL	0.189	*Y* = 3.053 − 0.051^***^ NL	0.246	*Y* = 2.318 − 0.027^***^ NL	0.236	*Y* = 2.488 − 0.029^***^ NL	0.504	*Y* = 3.059 − 0.042^***^ NL
Infructescence height	DL	0.194	*Y* = 3.894 − 0.024^***^ DL	0.046	*Y* = 2.456 − 0.010^***^ DL	0.125	*Y* = 3.484 − 0.024^***^ DL	0.270	*Y* = 4.390 − 0.033^***^ DL
	NL × DL	0.236	*Y* = 2.839 − 4.9.10^−4^^***^ NL × DL	0.234	*Y* = 2.179 − 2.5.10^−4^^***^ NL × DL	0.273	*Y* = 2.440 − 2.4.10^−4^^***^ NL × DL	0.515	*Y* = 2.836 − 4.2.10^−4^^***^ NL × DL
Number of fruitlets	NL	0.284	*Y* = 30.063 + 2.182^***^ NL	0.374	*Y* = 53.249 + 1.607^***^ NL	0.363	*Y* = 48.722 + 1.380^***^ NL	0.446	*Y* = 45.637 + 1.604^***^ NL
	DL	0.365	*Y* = −15.813 + 1.147^***^ DL	0.273	*Y* = −0.065 + 1.077^***^ DL	0.165	*Y* = 7.625 + 1.069^***^ DL	0.354	*Y* = −27.156 + 1.519^***^ DL
	NL × DL	0.406	*Y* = 36.252 + 0.022^***^ NL × DL	0.445	*Y* = 57.431 + 0.016^***^ NL × DL	0.412	*Y* = 51.271 + 0.016^***^ NL × DL	0.507	*Y* = 51.874 + 0.017^***^ NL × DL
**INTERNAL QUALITY ATTRIBUTES**									
Total soluble solids	NL	0.004	*Y* = 14.270 + 0.017^NS^NL	0.017	*Y* = 15.236 − 0.020^*^ NL	0.016	*Y* = 10.772 + 0.022^*^ NL	0.016	*Y* = 10.750 + 0.019^*^ NL
	DL	0.045	*Y* = 13.016 + 0.019^**^ DL	0.000	*Y* = 14.577 + 0.002^NS^DL	0.136	*Y* = 6.459 + 0.062^***^ DL	0.000	*Y* = 10.928 + 0.005^NS^DL
	NL × DL	0.018	*Y* = 14.174 + 2.4.10^−4^^*^ NL × DL	0.013	*Y* = 15.106 − 1.6.10^−4^^*^ NL × DL	0.052	*Y* = 10.465 + 3.8.10^−4^^***^ NL × DL	0.012	*Y* = 10.911 + 1.7.10^−4NS^NL × DL
Juice pH	NL	0.107	*Y* = 3.349 + 0.012^***^ NL	0.002	*Y* = 3.752 − 0.002^NS^NL	0.200	*Y* = 3.274 + 0.006^***^ NL	0.067	*Y* = 3.309 + 0.004^***^ NL
	DL	0.110	*Y* = 3.154 + 0.006^***^ DL	0.005	*Y* = 3.873 − 0.002^NS^DL	0.083	*Y* = 3.102 + 0.005^***^ DL	0.037	*Y* = 3.188 + 0.003^**^ DL
	NL × DL	0.139	*Y* = −3.395 + 1.2.10^−4^^***^ NL × DL	0.006	*Y* = 3.754 − 2.5.10^−5NS^NL × DL	0.224	*Y* = 3.280 + 7.2.10^−5^^***^ NL × DL	0.068	*Y* = 3.328 + 3.5.10^−5^^***^ NL × DL
Flesh translucency^c^	NL	0.039	*Y* = 2.249 + 0.081^**^ NL	0.199	*Y* = 1.704 + 0.119^***^ NL	0.000	*Y* = 1.759 + 0.024^NS^NL	0.041	*Y* = 1.494 + 0.046^**^ NL
	DL	0.007	*Y* = 2.521 + 0.019^NS^DL	0.042	*Y* = 0.901 + 0.044^**^ DL	0.051	*Y* = −4.982 + 0.094^***^ DL	0.048	*Y* = −1.295 + 0.052^***^ DL
	NL × DL	0.032	*Y* = 2.871 + 0.001^**^ NL × DL	0.197	*Y* = 2.260 + 0.001^***^ NL × DL	0.011	*Y* = 1.298 + 4.7.10^−4NS^NL × DL	0.051	*Y* = 1.634 + 0.001^***^ NL × DL

* Significant at the 0.05 probability level;

** Significant at the 0.01 probability level;

*** Significant at the 0.001 probability level;

a NL, number of functional leaves at flower induction;

b DL, D-leaf length at flower induction;

c *Regression was based on square root transformed data*.

The cross product NL × DL was also significantly positively associated with the fruit height and the association was very clear for cv. Sugarloaf (Table 3; Figures 1D1,D2); for cv. Smooth Cayenne, this association was poorer although significant in both experiments (Table 3; Figures 1D3,D4). Of the attributes underlying fruit height, the infructescence height also increased with an increase in NL × DL in all experiments (Figures 1E1–E4), but the crown height was differently related to NL × DL in the two cultivars; for cv. Sugarloaf a weak positive association was found to be significant only in one of the two experiments whereas a negative association was found in both Smooth Cayenne experiments (Table 3). As for crown weight, crown height showed a better association with DL than with NL × DL, but for cv. Sugarloaf only. For cv. Smooth Cayenne, the negative association between the initial plant vigor and crown height was even clearer for NL than for NL × DL in one experiment (Table 3).

The cross product NL × DL was significantly negatively associated with the ratio crown height: infructescence height (Table 3; Figures 1G1–G4) in all experiments.

Figures showing the associations of the external quality attributes with NL and DL can be found in the supplementary material (Supplementary Figures S1,2).

### Associations between plant vigor at the time of artificial flower induction and internal fruit quality attributes at harvest

**Figure 2:**
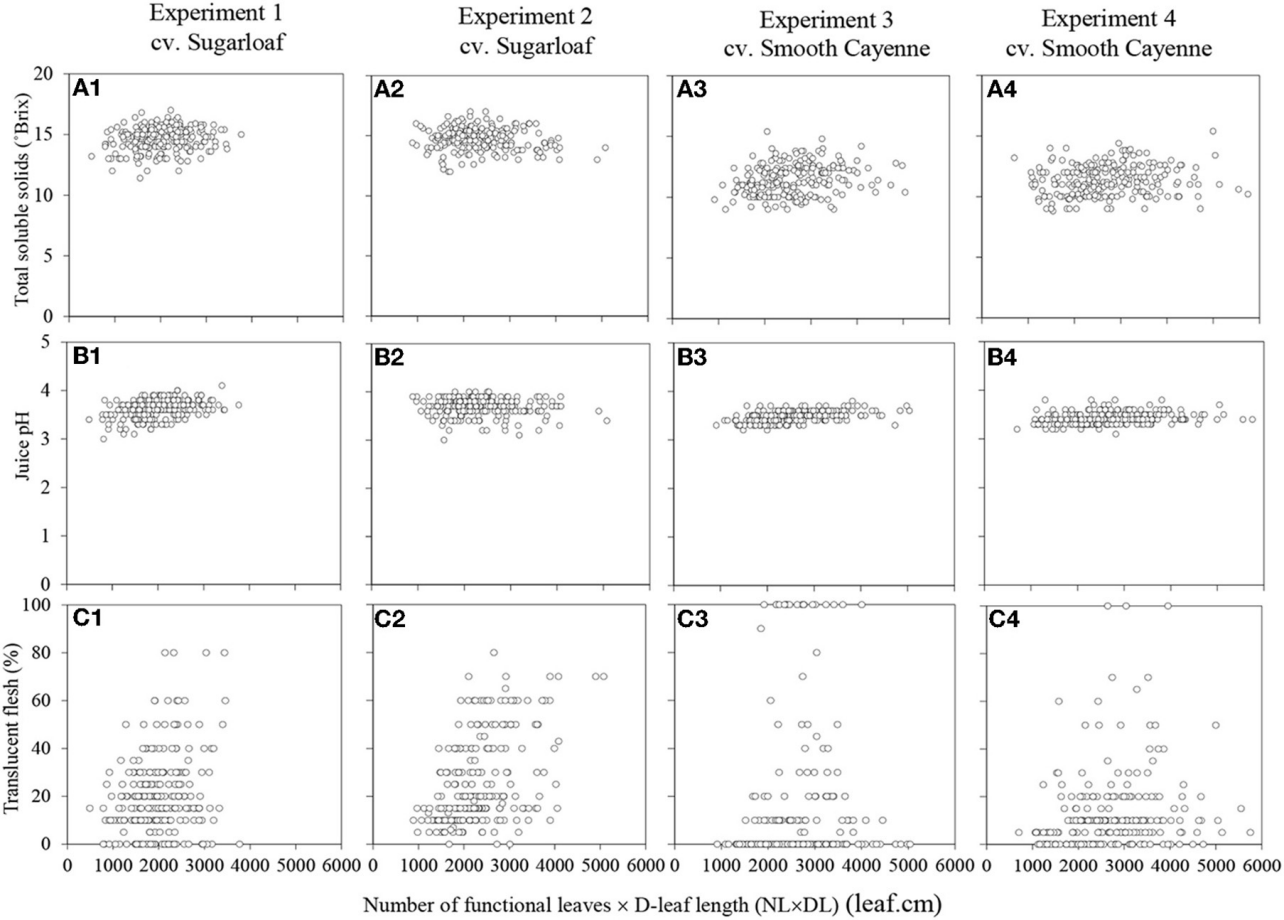
**Associations between the number of functional leaves × the D-leaf length (NL × DL) on the internal fruit quality attributes in Experiments 1 (A1–C1) and 2 (A2–C2) (cv. Sugarloaf) and Experiments 3 (A3–C3) and 4 (A4–C4) (cv. Smooth Cayenne)**.

The plant vigor variates at the time of artificial flower induction were not or only weakly associated with the TSS, juice pH and translucency of the fruits at harvest (Table 3; Figure 2 for associations with NL × DL). Figures showing the associations with NL and DL can be found in the supplementary material (Supplementary Figures S3 and S4).

Weak but significant associations between at least one of the vigor variates and TSS were found in all experiments, but these associations were positive in three experiments and negative in one experiment, and consequently not consistent across experiments (Table 3).

For cv. Smooth Cayenne, the cross product NL × DL was the strongest vigor variate to be weakly, but consistently positively associated with juice pH (Table 3; Figures 2B3,B4). For cv. Sugarloaf the same results were found in Experiment 1 (Table 3; Figure 2B1), whereas in Experiment 2 no significant associations were found between any of the vigor variates and juice pH (Table 3; Figure 2B2; Supplementary Figures S3-B2, S4-B2 in Supplementary material).

No consistent associations were found between the vigor variates and flesh translucency for cv. Smooth Cayenne (Table 3). For cv. Sugarloaf, NL was the strongest vigor variate to be weakly but consistently associated with flesh translucency (Table 3; Supplementary Figures S3-C1, C2 in Supplementary material).

### Influence of side shoot production on the association between initial plant vigor and fruit quality at harvest

#### Production of side shoots

The type of side shoots (slips, hapas, and suckers) produced at harvest time was not the same for the two pineapple cultivars and differed across the two experiments per cultivar. Sugarloaf produced mainly slips; the number of plants producing slips was higher in Experiment 2 than in Experiment 1 (Table 4). No slips were observed in cv. Smooth Cayenne. Only very few plants produced hapas in both cultivars (Table 4) and none had produced suckers at harvest time (Table 4). Based on these results, only Experiment 2 was used to test whether the number and/or the weight of the slips produced accounted additionally for fruit quality heterogeneity.

**Table 4:** **Number of plants that produced a certain type of side shoot in the four experiments, cvs Sugarloaf and Smooth Cayenne**.

**Type of side shoots**	**Cv. Sugarloaf**		**Cv. Smooth Cayenne**	
	**Experiment 1 (*n* = 240)**	**Experiment 2 (*n* = 240)**	**Experiment 3 (*n* = 227)**	**Experiment 4 (*n* = 234)**
Slips	13	182	0	0
Hapas	1	5	2	5
Suckers	0	0	0	0

#### Number or weight of slips accounting for the fruit quality heterogeneity

Pearson's correlation coefficient revealed that there was a strong and positive correlation between the different plant vigor variates and the number and weight of slips at harvest (Table 5). However, since the correlation coefficients were not above 0.80, we concluded that there was no multiple colinearity. Therefore, the number or weight of the slips was added as additional explanatory variate to the linear regression models in Table 3.

**Table 5:** **Pearson correlation coefficient (*r*) between plant vigor variates at the time of artificial flower induction and the number and weight of slips at harvest across individual plants in Experiment 2, cv. Sugarloaf (*n* = 240)**.

**Plant vigor variate**	**Slip number**	**Slip weight**
Number of functional leaves (NL)	0.571^***^	0.576^***^
D-leaf length (DL)	0.542^***^	0.570^***^
Cross product (NL × DL)	0.650^***^	0.671^***^

*** *Significant at 0.001 probability level*.

The addition of the number of slips to the regression models did not significantly increase the explanation of the variability (adjusted *R*^2^) in the external and internal quality attributes (Table 6). The weight of the slips significantly increased the explained variability in fruit weight, infructescence weight and the fruit height. Higher slip weight was associated with higher fruit weight, infructescence weight and fruit height (Table 6).

**Table 6:** **Multiple regression models showing the association between the strongest plant vigor variate at the time of flower induction plus slip weight or number (explanatory variates) and external fruit quality attributes at harvest (response variates) in Experiment 2, cv. Sugarloaf (*n* = 240)**.

**Fruit quality attribute at harvest**	**Explanatory variates**	**Experiment 2**		
		***R*^2^ adj**.	***P*-value for significance in *F* change^a^**	**Equation**
**EXTERNAL QUALITY ATTRIBUTES**				
Fruit weight	NL^b^ × DL^c^ + SN^d^	0.688	0.085	*Y* = 0.307 + 3.62.10^−4^^***^ NL × DL + 0.011^NS^ SN
	NL × DL + SW^e^	0.690	0.035^*^	*Y* = 0.324 + 3.55.10^−4^^***^ NL× DL + 1.43.10^−4^^*^ SW
Infructescence weight	NL × DL + SN	0.682	0.064	*Y* = 0.135 + 3.56.10^−4^^***^ NL× DL + 0.012^NS^ SN
	NL × DL + SW	0.683	0.038^*^	*Y* = 0.150 + 3.51.10^−4^^***^NL× DL + 1.42.10^−4^^*^ SW
Crown weight	DL + SN	0.028	0.166	*Y* = 0.109 + 0.001** DL − 0.001^NS^ SN
	DL + SW	0.022	0.476	*Y* = 0.116 + 0.001*DL − 7.92.10^−6NS^ SW
Fruit height	NL × DL + SN	0.397	0.062	*Y* = 32.312 + 0.003^***^ NL × DL + 0.206^NS^ SN
	NL × DL + SW	0.402	0.019^*^	*Y* = 32.666 + 0.003^***^ NL × DL + 0.003* SW
Infructescence height	NL × DL + SN	0.587	0.091	*Y* = 8.841 + 0.003^***^ NL × DL + 0.107^NS^ SN
	NL × DL + SW	0.588	0.078	*Y* = 8.946 + 0.003^***^ NL × DL + 0.001^NS^ SW
Crown height	DL + SN	0.095	0.367	*Y* = 12.441 + 0.140^***^ DL − 0.087^NS^ SN
	DL + SW	0.093	0.510	*Y* = 12.609 + 0.137^***^ DL − 0.001^NS^ SW
Ratio crown height:	NL × DL + SN	0.229	0.776	*Y* = 2.171 − 2.40.10^−4^^***^ NL × DL − 0.003^NS^ SN
Infructescence height	NL × DL + SW	0.229	0.953	*Y* = 2.182 − 2.50.10^−4^^***^ NL × DL + 9.22.10^−6NS^ SW
Number of fruitlets	NL × DL + SN	0.448	0.087	*Y* = 59.048 + 0.015^***^ NL × DL + 0.638^NS^ SN
	NL × DL + SW	0.450	0.138	*Y* = 59.921 + 0.014^***^ NL × DL + 0.008^NS^ SW
**INTERNAL QUALITY ATTRIBUTES**				
Total soluble solids	NL + SN	0.022	0.145	*Y* = 15.350 − 0.028** NL + 0.040^NS^ SN
	NL + SW	0.013	0.958	*Y* = 15.241 − 0.020^NS^ NL + 1.52.10^−5NS^ SW
Flesh translucency	NL + SN	0.200	0.250	*Y* = 1.865 + 0.107^***^ NL + 0.056^NS^ SN
	NL + SW	0.203	0.131	*Y* = 1.963 + 0.103^***^ NL + 0.001^NS^ SW

* Significant at the 0.05 probability level;

a Significance of the F change after adding SN or SW to the regression model;

b NL, number of functional leaves at flower induction;

c DL, D-leaf length at flower induction;

d SN, slip number;

e *SW, slip weight*.

## Discussion

### Plant vigor at the time of artificial flower induction and external fruit quality at harvest

Our data show that in the pineapple crops, most of the external quality attributes of the fruit at harvest were significantly and positively associated with the initial vigor of the plant at the moment of artificial flower induction (Table 3).

Differences in initial plant vigor accounted for a high proportion of the variation in fruit weight. Comparing the three vigor variates, the highest proportion of the heterogeneity in fruit weight was explained by NL × DL (Table 3; Figures 1A1–A4). The association between the NL × DL and the fruitlets number and the fruit weight at harvest was positive. Reasons explaining this are likely that out of the three vigor variates, NL × DL would be best related to leaf area, and that higher values of the NL × DL at the time of artificial flower induction thus would indicate a higher leaf area and consequently a higher photosynthetic capacity and amount of assimilates available in a plant at the time of artificial flower induction i.e., at the end of the vegetative phase. Since the production of new normal leaves ceases once flowering is induced (Bartholomew and Malézieux, 1994), the available assimilates at the flower induction time that were allocated to the roots and leaves, now additionally are partitioned to the new sinks, i.e., the infructescence, crown and peduncle. Earlier studies showed that a large proportion of assimilates is allocated to the infructescence and the crown (Marler, 2011b). This means that the more assimilates are available at flower induction, the higher would be the fruit weight. The association of fruit weight with plant vigor at flower initiation shows the importance of the development stage and morphology of the plants at flower induction for final fruit quality, and is consistent with experiments in which later flower induction increased fruit weight in whole crops (Mitchell, 1962; Bartholomew et al., 2003) and in individual plants (Van Overbeek, 1946).

Our data show that the positive association between the initial plant vigor and later fruit weight was mainly due to an effect on the infructescence weight whereas the effect on the crown was much smaller and only consistently significant for one vigor variate (Table 3; Figures 1C1–C4; Supplementary Figures S1-C1 to C4 and S2-C1 to C4 in Supplementary material). Such differences in the effect on the infructescence and crown could probably be explained by the differences in timing of their development. The initiation of the florets may have continued longer in infructescences bearing more florets, which may have delayed the onset of crown formation.

Each floret differentiates into one fruitlet. Our results revealed that in all experiments, all plant vigor variates are positively associated with the number of fruitlets at harvest (Figures 1H1–H4; Supplementary Figures S1-H1 to H4 and S2-H1 to H4) indicating that in vigorous plants more florets were able to develop into fruitlets. As with fruit weight, NL × DL was the plant vigor variate explaining the largest proportion of variation in number of fruitlets. After flower induction, pineapple plants show an increase of the width of the apex (Wee and Rao, 1979) which bears the florets. Thus, more assimilates available—plants with high NL × DL—would lead to high volume increase of the apex and consequently high number of florets that will differentiate into fruitlets.

Considering the fruit height, it was found that the association between NL × DL and the fruit height was strong in the experiments with cv. Sugarloaf (*R*^2^ = 0.402 and 0.390 in Experiments 1 and 2, respectively) and significant but much weaker in the experiments with cv. Smooth Cayenne (*R*^2^ = 0.060 and 0.024 in Experiments 3 and 4, respectively) (Table 3; Figures 1D1–D4). These differences were due to the differences between cultivars in the associations between NL × DL and fruit height components: infructescence height and crown height. The former was positive for both cultivars, but the association between NL × DL and crown height was positive for cv. Sugarloaf (Table 3; Figures 1F1,F2) and negative for cv. Smooth Cayenne (Table 3; Figures 1F3,F4). This means that for cv. Smooth Cayenne, more vigorous plants produce fruits with a shorter crown (Figures 1F3,F4) lowering then the total fruit height, hence the poor association observed between the NL × DL and the fruit height at harvest for cv. Smooth Cayenne. This is also in line with the significantly negative correlations between the infructescence height and the crown height for cv. Smooth Cayenne (Supplementary Tables S3, S4 in Supplementary material).

The negative associations between NL × DL and the ratio crown height: infructescence height (Table 1; Supplementary Tables S1, S2, S3, and S4 in Supplementary material; Figure 1-G1 to G4) follow logically from the clear increase in infructescence height with increase in NL × DL (Figures 1E1–E4) combined with the poor and negative association between the initial plant vigor and the crown height. Reasons for such differences are described above.

These associations between the plant vigor at artificial flower induction and the external fruit quality attributes suggest there is a good chance of decreasing the heterogeneity in fruit quality within a lot by increasing the uniformity of the crop at the moment of flower induction. This could be achieved before and after planting. Uniform soil conditions will be the basis for uniform growth after planting. Also selecting more uniform planting material will increase uniformity; using specific clonal planting material production, for example as suggested by Agogbua and Osuji (2011) in Nigeria, could be an option to not only provide uniform planting material but also enough planting material to cope with the current practice of mixing planting material at planting. After planting, the selective application of extra fertilizers to the least developed plants may increase their vigor so that they reach the vigorous plants before the moment of artificial flower induction. In theory, also pruning leaves from the most advanced plants may increase uniformity by decreasing the vigor of the best plants, but this may also lower the average fruit quality. Also, selective flower induction, i.e., induction of the least and most vigorous plants at different times, could help improve the uniformity of the fruit at harvesting time.

### Plant vigor at the time of artificial flower induction and internal fruit quality at harvest

Heterogeneity in pineapple taste is also a problem in the pineapple supply chain (Fassinou Hotegni et al., 2014). In the present paper, TSS and juice pH were assessed to represent taste. Our findings indicated that the variation in TTS and especially in pH were very small compared to those in fruit and infructescence weight. There were no clear associations between the initial plant vigor and TSS, juice pH or flesh translucency since the results were not consistent across experiments. Such results are in line with the idea that fruit ripening and maturation—affecting TSS and juice pH—occur autonomous in proportion to the fruit size established, and in relation to time and external conditions. However, for the flesh translucency, results showed a consistent positive correlation between translucency and TSS in the experiments with Smooth Cayenne (Supplementary Tables S3, S4 in Supplementary material). These results on flesh translucency in cv. Smooth Cayenne confirm the findings of Chen and Paull (2001), that translucency is affected by sugar concentration at harvest time.

### Cultivar differences in heterogeneity in external and internal quality at harvest

In this study, the experiments with cv. Smooth Cayenne showed a higher variation than the experiments with cv. Sugarloaf in some external quality attributes and internal quality attributes (Table 2). We attribute most of these differences to genotypic differences and differences in the cultivation practices of these cultivars, although the differences between experiments also might be affected by the location and season. The high variation in the crown weight and height in cv. Smooth Cayenne compared to cv. Sugarloaf (Table 2) might originate in part from the diverse planting material; mixtures of hapas and suckers were used in cv. Smooth Cayenne planting while only slips were used in cv. Sugarloaf planting. It is well-known that plants grown from suckers initiate fruits earlier than plants grown from hapas (Bartholomew et al., 2003); so variation would exist in the growth of the two types of planting material. In our study, variation in plant vigor variates at flower induction was similar for both cultivars. Therefore, variation in growth of the hapas and suckers expresses itself later during the generative phase increasing variation in crown weight and height in cv. Smooth Cayenne and suggesting a relationship between the type of planting material used and the morphology of the fruit produced. The higher variation in the ratio crown: infructescence height in cv. Smooth Cayenne than in cv. Sugarloaf was certainly the consequence of a higher variation in crown height and opposite associations between plant vigor and crown height, and plant vigor and infructescence height (Supplementary Tables S3,S4 in Supplementary material).

When considering the internal quality attributes, variation in TSS and translucency was higher in cv. Smooth Cayenne than in cv. Sugarloaf while for the variation in juice pH the opposite was observed. Differences in variation in TSS between the two cultivars might be due to maturity synchronization practices in cv. Smooth Cayenne which might increase variation in TSS. In pineapple fruits, at 2 weeks before the ripening of the fruit, the TSS increases until the harvest (Singleton and Gortner, 1965); when maturity is synchronized by applying Ethrel on the skin of the fruits—at different stages of natural ripening process (different TSS)—degreening of the shell is accelerated artificially (Smith, 1991). Then, the variation in TSS will be higher in cv. Smooth Cayenne when compared to cv. Sugarloaf where no maturity was synchronized. Higher variation in flesh translucency in cv. Smooth Cayenne might be due to the high variation in TSS; TSS and translucency are positively associated in cv. Smooth Cayenne as shown in Supplementary Tables S3,S4 in the Supplementary material.

### Slip weight effect on fruit quality heterogeneity at harvest

The weight of slips but not the number of slips accounted for an extra part of the variation in fruit weight, infructescence weight and fruit height in addition to the effect related to the initial plant vigor (Table 6). This effect of the slip weight was positive (Table 6). Differences in fruit weight, infructescence weight, and the height of the fruit thus may not originate only from differences in initial plant vigor but also to a small extent from differences in the weight of slips produced. This might be the result of transfer of assimilates from the slips to the fruit (Marler, 2011a). Slips are composed of leaves and the slip weight will give a better idea of the photosynthetic capacity of the slips than the slip number. A better understanding of the role and the determinants of the variation of slip production within a crop and between crops would help to improve fruit weight, infructescence weight, and fruit height.

## Conclusions and implications

The heterogeneity in fruit weight, infructescence weight and height, the number of fruitlets, and ratio crown height: infructescence height in pineapple crops is a direct consequence of the heterogeneity in plant vigor at the time of artificial flower induction of these crops. Among the plant vigor variates the cross product NL × DL was the vigor variate explaining the highest proportion (up to 68.7%) of the variance in fruit weight; that effect was mainly on the infructescence weight and less or not on the crown weight. In addition to the plant vigor variates, slip weight also accounted for variation in fruit weight, infructescence weight and fruit height. Plant vigor at flower induction was weakly and not consistently associated with TSS, juice pH, and the flesh translucency. Differences existed between experiments with different cultivars; a higher variation in crown weight, crown height and ratio crown: infructescence height, TSS, and translucency but a lower variation in pH was observed in cv. Smooth Cayenne than in cv. Sugarloaf.

Results from this study are important to design agronomic tools to get a more uniform fruit weight quality at harvest without reducing the overall quality. Achieving a more uniform crop with regards to plant vigor—especially NL × DL—at flower induction would reduce the fruit quality heterogeneity, especially the external fruit quality, at harvest. This could probably be achieved by reducing heterogeneity in planting material at planting through the use of uniform planting material in terms of type (hapas or suckers in cv. Smooth Cayenne) and weight. The use of clonal propagation could also be an option to increase uniformity in planting material at planting time. Other options could be the selective application of extra fertilizers to the least developed plants to increase their vigor or selective induction of flowering of plants depending on their vigor.

### Conflict of interest statement

The Associate Editor Olaf Van Kooten declares that, despite being affiliated to the same institution as the authors V. Nicodème Fassinou Hotegni, Willemien J. M. Lommen and Paul C. Struik, the review process was handled objectively and no conflict of interest exists. The authors declare that the research was conducted in the absence of any commercial or financial relationships that could be construed as a potential conflict of interest.

## Abbreviations

CV: Coefficient of variation
NL: Number of functional leaves
DL: D-leaf length
TSS: Total soluble solids.
