# Supplementary material for: Heterogeneity in pineapple fruit quality results from plant heterogeneity at flower induction
Source: Front Plant Sci. 2014 Dec 9;5:670. doi: 10.3389/fpls.2014.00670 (PMC4260489; doi:10.3389/fpls.2014.00670)
Supplement: Supplementary file 1 [file DataSheet1.PDF]

Supplementary Material

Heterogeneity in Pineapple Fruit Quality Results from Plant Heterogeneity at Flower Induction

V.N. Fassinou Hotegni[1,2], W.J.M. Lommen[1*], E.K. Agbossou[2] and P.C. Struik[1]

[1] Centre for Crop Systems Analysis, Wageningen University, Wageningen, the Netherlands
[2] Faculté des Sciences Agronomiques, Université d'Abomey Calavi, Cotonou, Benin,

***Correspondence:** Dr. Willemien J. M. Lommen, Centre for Crop Systems Analysis, Wageningen University, 6708 PB Wageningen, the Netherlands
willemien.lommen@wur.nl

1. Supplementary material

Tables S1, S2, S3 and S4 and Figures S1, S2, S3 and S4.

Table S1 Correlation matrix showing Pearson correlation coefficient (*r*) between the plant vigor variates at the time of artificial flower induction and the different quality attributes at harvest for Experiment 1, cv. Sugarloaf (n=240)

	NL	DL	NL×DL	Fruit weight	IW	Crown weight	Fruit height	IH	Crown height	Ra	Number of fruitlets	TSS	Juice pH	Trans-lucency
NL[a]	1	0.483***	0.928***	0.689***	0.690***	0.287***	0.505***	0.582***	0.118	-0.438***	0.536***	0.088	0.333***	0.207***
DL[b]		1	0.761***	0.709***	0.696***	0.381***	0.636***	0.652***	0.226***	-0.444***	0.606***	0.222***	0.337***	0.105
NL×DL			1	0.804***	0.799***	0.369***	0.636***	0.692***	0.188**	-0.489***	0.639***	0.149*	0.377***	0.189**
Fruit weight				1	0.991***	0.487***	0.751***	0.856***	0.184**	-0.594***	0.720***	0.098	0.289***	0.176**
IW[c]					1	0.363***	0.700***	0.871***	0.101	-0.640***	0.743***	0.077	0.278***	0.184**
Crown weight						1	0.646***	0.270***	0.608***	0.039	0.157*	0.175**	0.191**	0.024
Fruit height							1	0.669***	0.698***	-0.214***	0.481***	0.079	0.236***	0.132*
IH[d]								1	-0.065	-0.830***	0.778***	0.030	0.217**	0.132*
Crown height									1	0.512***	-0.103	0.077	0.108	0.051
Ra[e]										1	-0.665***	-0.023	-0.131*	-0.099
Number of fruitlets											1	-0.050	0.181**	0.209**
TSS[f]												1	0.392***	-0.126
Juice pH													1	0.005
Translucency														1

* Significant at the 0.05 probability level; ** Significant at the 0.01 probability level; *** Significant at the 0.001 probability level; [a]: NL, number of functional leaves at flower induction; [b]: DL, D-leaf length at flower induction; [c]: IW, infructescence weight; [d]: IH, infructescence height; [e]: Ra, ratio crown height: infructescence height; [f]: TSS, total soluble solids

Table S2 Correlation matrix showing Pearson correlation coefficient (*r*) between the plant vigor variates at the time of artificial flower induction and the different quality attributes for Experiment 2, cv. Sugarloaf (n=240)

	NL	DL	NL×DL	Fruit weight	IW	Crown weight	Fruit height	IH	Crown height	Ra	Number of fruitlets	TSS	Juice pH	Trans-lucency
NL[a]	1	0.386***	0.960***	0.787***	0.787***	0.049	0.529***	0.716***	0.044	-0.499***	0.614***	-0.146*	-0.080	0.450***
DL[b]		1	0.619***	0.534***	0.519***	0.167**	0.616***	0.530***	0.315***	-0.224***	0.526***	0.016	-0.095	0.213***
NL×DL			1	0.829***	0.825***	0.085	0.627***	0.765***	0.124	-0.487***	0.669***	-0.132*	-0.100	0.447***
Fruit weight				1	0.995***	0.104	0.679***	0.883***	0.088	-0.592***	0.743***	-0.190**	-0.099	0.541***
IW[c]					1	0.002	0.630***	0.900***	0.011	-0.649***	0.760***	-0.204***	-0.101	0.527***
Crown weight						1	0.515***	-0.123	0.756***	0.518***	-0.125	0.138*	0.020	0.171**
Fruit height							1	0.613***	0.727***	-0.037	0.557***	-0.031	-0.152*	0.424***
IH[d]								1	-0.097	-0.789***	0.821***	-0.202**	-0.148*	0.371***
Crown height									1	0.640***	-0.012	0.136*	-0.063	0.212**
Ra[e]										1	-0.644***	0.199**	0.049	-0.158*
Number of fruitlets											1	-0.146*	-0.085	0.318***
TSS[f]												1	0.467***	0.029
Juice pH													1	0.134*
Translucency														1

* Significant at the 0.05 probability level; ** Significant at the 0.01 probability level; *** Significant at the 0.001 probability level; [a]: NL, number of functional leaves at flower induction; [b]: DL, D-leaf length at flower induction; [c]: IW, infructescence weight; [d]: IH, infructescence height; [e]: Ra, ratio crown height: infructescence height; [f]: TSS, total soluble solids

Table S3 Correlation matrix showing Pearson correlation coefficient (*r*) between the plant vigor variates at the time of artificial flower induction and the different quality attributes for Experiment 3, cv. Smooth Cayenne (n=227)

	NL	DL	NL×DL	Fruit weight	IW	Crown weight	Fruit height	IH	Crown height	Ra	Number of fruitlets	TSS	Juice pH	Trans-lucency
NL[a]	1	0.283***	0.959***	0.643***	0.642***	0.164*	0.220***	0.585***	-0.160*	-0.489***	0.605***	0.144*	0.451***	0.068
DL[b]		1	0.533***	0.447***	0.421***	0.202**	0.197**	0.425**	-0.076	-0.359***	0.410***	0.373***	0.295***	0.236***
NL×DL			1	0.685***	0.678***	0.195**	0.253***	0.635***	-0.158*	-0.525***	0.644***	0.236***	0.477***	0.124
Fruit weight				1	0.962***	0.380***	0.403***	0.831***	-0.130*	-0.619***	0.820***	0.294***	0.345***	0.348***
IW[c]					1	0.114	0.242***	0.838***	-0.307***	-0.719***	0.826***	0.267***	0.414***	0.380***
Crown weight						1	0.653***	0.184**	0.569***	-0.185**	0.186**	0.165*	-0.149*	-0.021
Fruit height							1	0.402***	0.789***	0.205**	0.289***	0.030	-0.091	0.005
IH[d]								1	-0.245***	-0.786***	0.754***	0.231***	0.347***	0.257***
Crown height									1	0.744***	-0.200**	-0.123	-0.331***	-0.161*
Ra[e]										1	-0.622***	-0.242***	-0.418***	-0.270***
Number of fruitlets											1	0.246***	0.392***	0.260***
TSS[f]												1	0.171**	0.308***
Juice pH													1	0.297***
Translucency														1

* Significant at the 0.05 probability level; ** Significant at the 0.01 probability level; *** Significant at the 0.001 probability level; [a]: NL, number of functional leaves at flower induction; [b]: DL, D-leaf length at flower induction; [c]: IW, infructescence weight; [d]: IH, infructescence height; [e]: Ra, ratio crown height: infructescence height; [f]: TSS, total soluble solids

Table S4 Correlation matrix showing Pearson correlation coefficient (*r*) between the plant vigor variates at the time of artificial flower induction and the different quality attributes for Experiment 4, cv. Smooth Cayenne (n=234)

	NL	DL	NL×DL	Fruit weight	IW	Crown weight	Fruit height	IH	Crown height	Ra	Number of fruitlets	TSS	Juice pH	Trans-lucency
NL[a]	1	0.491***	0.966***	0.646***	0.684***	0.025	0.106	0.697***	-0.417***	-0.712***	0.670***	0.143*	0.266***	0.213***
DL[b]		1	0.684***	0.547***	0.532***	0.236***	0.312***	0.595***	-0.125	-0.523***	0.597***	0.038	0.203**	0.227***
NL×DL			1	0.682***	0.710***	0.078	0.169**	0.736***	-0.381***	-0.719***	0.713***	0.128	0.269***	0.234***
Fruit weight				1	0.980***	0.391***	0.512***	0.924***	0.166*	-0.716***	0.828***	0.155*	0.411***	0.324***
IW[c]					1	0.199**	0.399***	0.942***	-0.298***	-0.781***	0.830***	0.159*	0.424***	0.310***
Crown weight						1	0.671***	0.193**	0.552***	0.082	0.236***	0.031	0.063	0.162*
Fruit height							1	0.416***	0.726***	0.056	0.388***	-0.076	0.085	0.189**
IH[d]								1	-0.324***	-0.852***	0.848***	0.131*	0.392***	0.304***
Crown height									1	0.703***	-0.238***	-0.178**	-0.208***	-0.033
Ra[e]										1	-0.737***	-0.195**	-0.364***	-0.259***
Number of fruitlets											1	0.153*	0.278***	0.316***
TSS[f]												1	0.033	0.152*
Juice pH													1	-0.146*
Translucency														1

* Significant at the 0.05 probability level; ** Significant at the 0.01 probability level; *** Significant at the 0.001 probability level; [a]: NL, number of functional leaves at flower induction; [b]: DL, D-leaf length at flower induction; [c]: IW, infructescence weight; [d]: IH, infructescence height; [e]: Ra, ratio crown height: infructescence height; [f]: TSS, total soluble solids

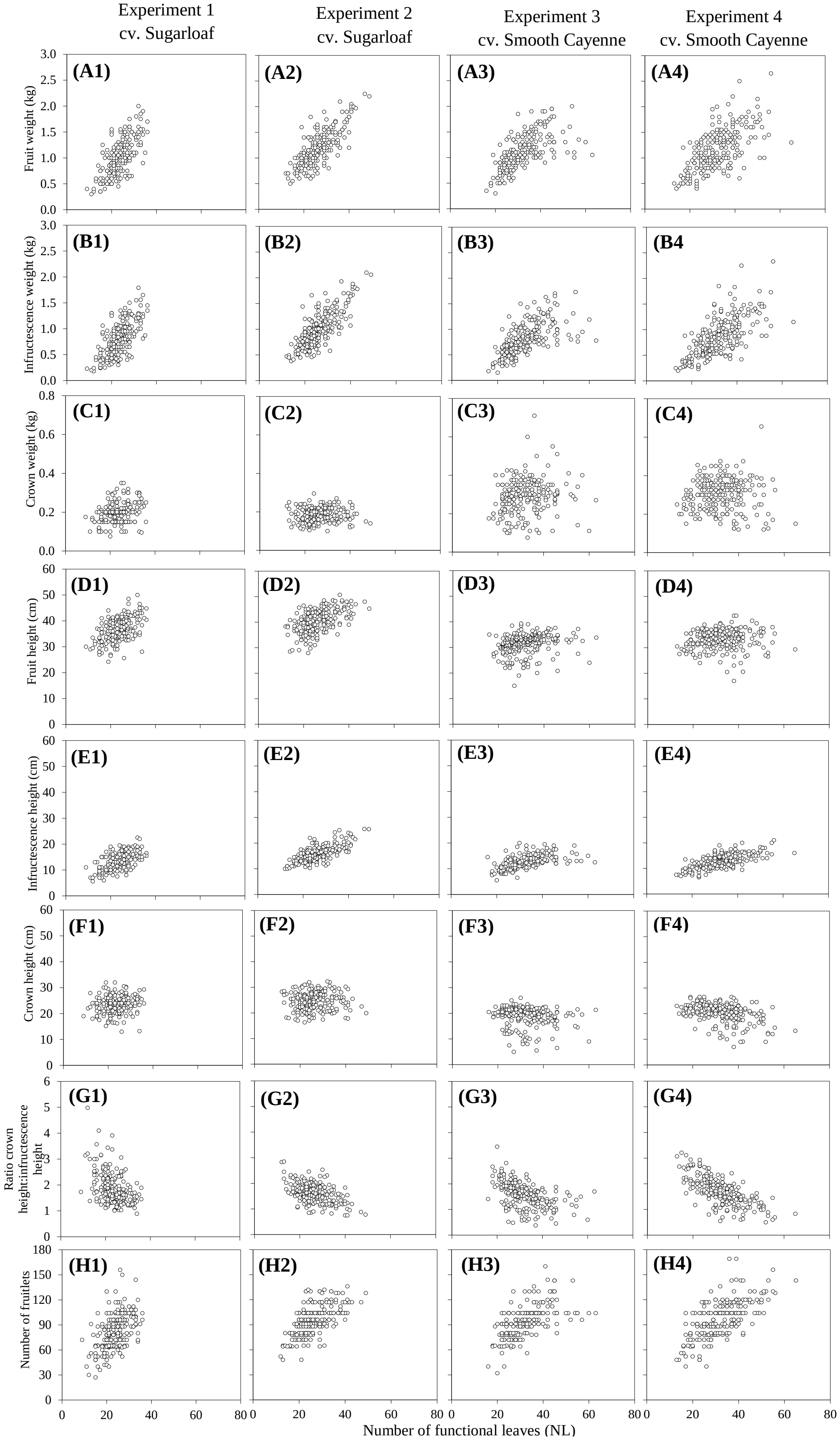

Figure S1 Association between the number of functional leaves (NL) and the external fruit quality attributes in Experiments 1 (A1 to H1) and 2 (A2 to H2) (cv. Sugarloaf) and Experiments 3 (A3 to H3) and 4 (A4 to H4) (cv. Smooth Cayenne)

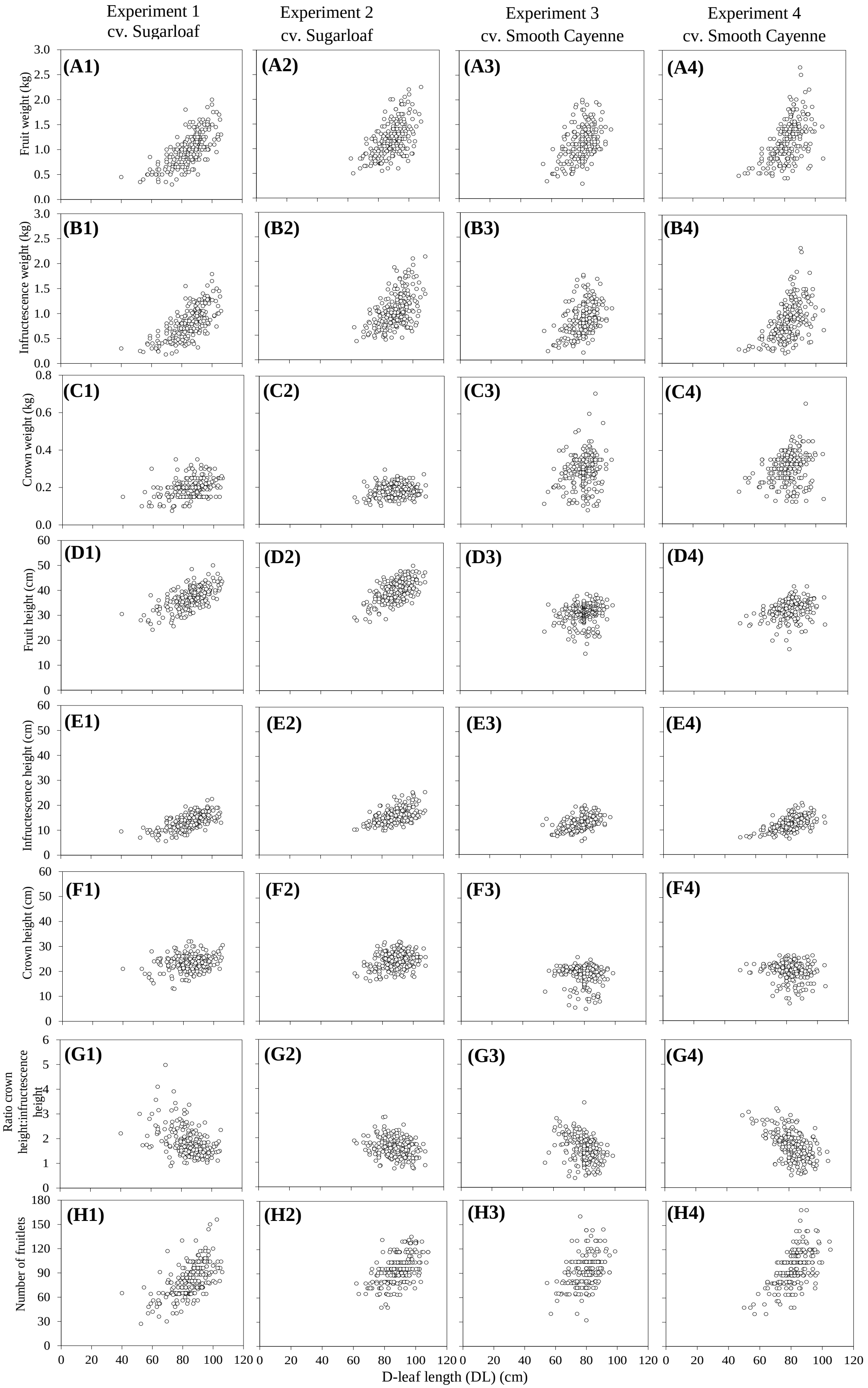

Figure S2 Association between the D-leaf length (DL) and the external fruit quality attributes in Experiments 1 (A1 to H1) and 2 (A2 to H2) (cv. Sugarloaf) and Experiments 3 (A3 to H3) and 4 (A4 to H4) (cv. Smooth Cayenne)

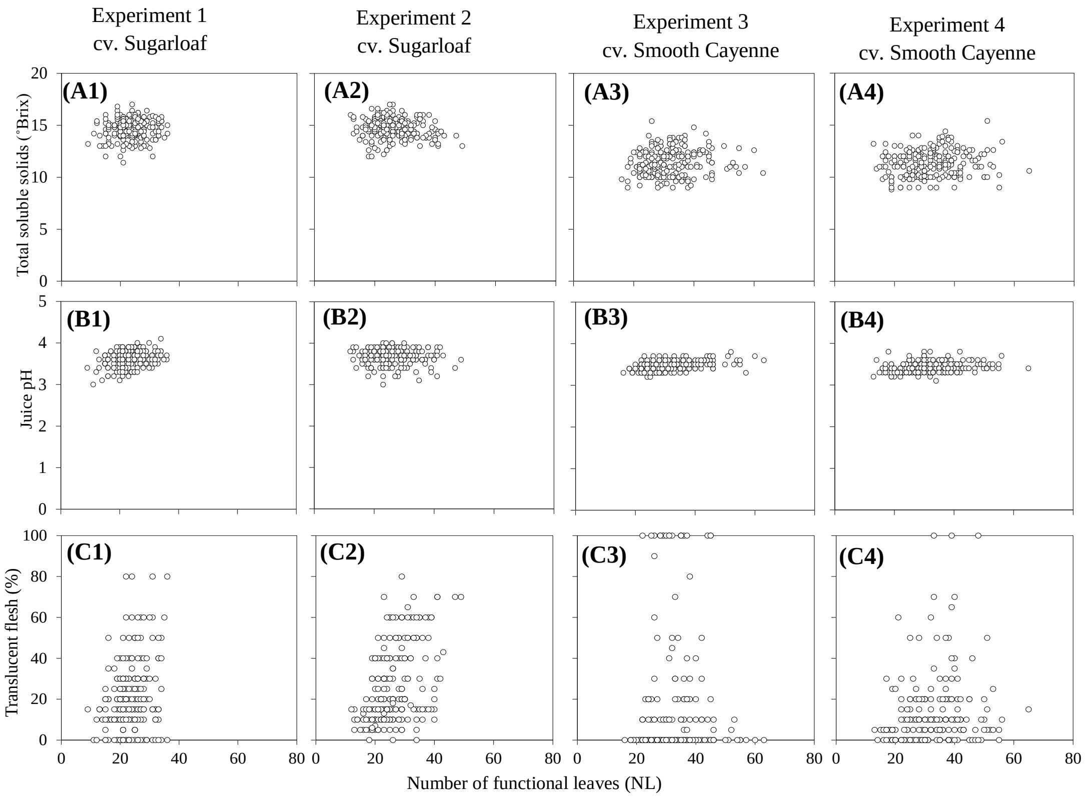

Figure S3 Association between the number of functional leaves (NL) and the internal fruit quality attributes in Experiments 1 (A1 to C1) and 2 (A2 to C2) (cv. Sugarloaf) and Experiments 3 (A3 to C3) and 4 (A4 to C4) (cv. Smooth Cayenne)

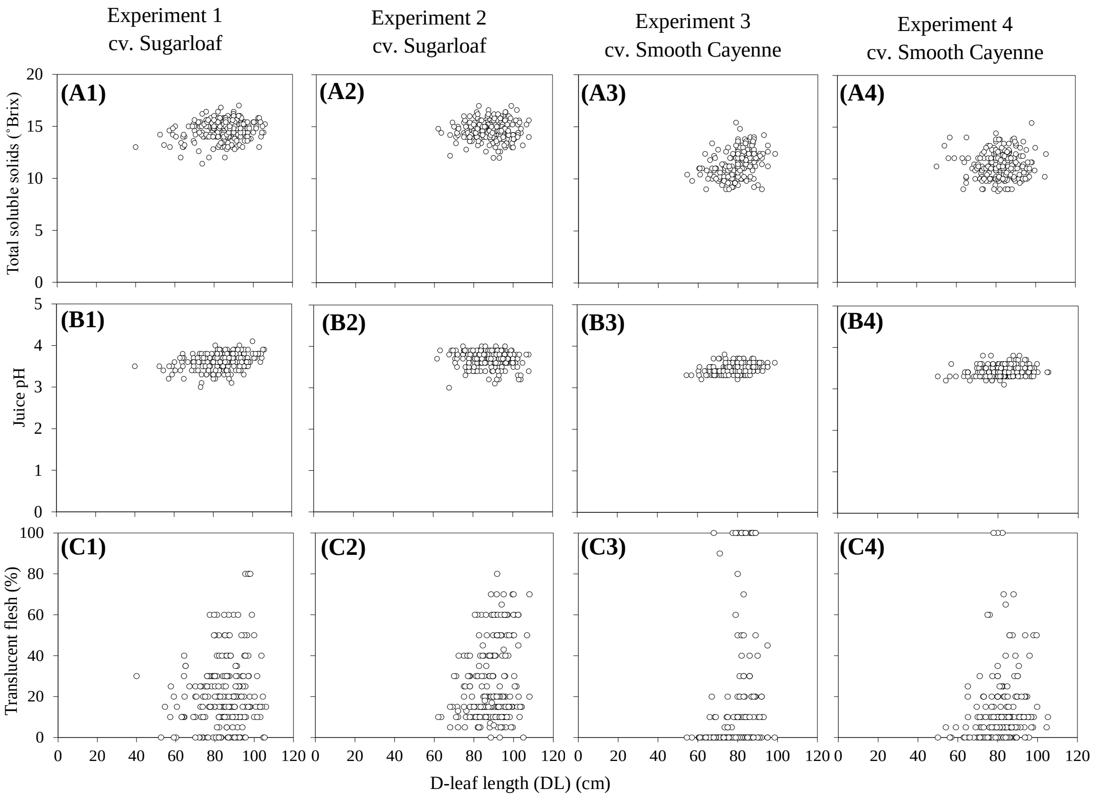

Figure S4 Association between the D-leaf length (DL) and the internal fruit quality attributes in Experiments 1 (A1 to C1) and 2 (A2 to C2) (cv. Sugarloaf) and Experiments 3 (A3 to C3) and 4 (A4 to C4) (cv. Smooth Cayenne)
